# Investigation of dosimetric variations of liver radiotherapy using deformable registration of planning CT and cone‐beam CT

**DOI:** 10.1002/acm2.12008

**Published:** 2016-12-05

**Authors:** Pu Huang, Gang Yu, Jinhu Chen, Changsheng Ma, Shaohua Qin, Yong Yin, Yueqiang Liang, Hongsheng Li, Dengwang Li

**Affiliations:** ^1^ Shandong Province Key Laboratory of Medical Physics and Image Processing Technology Institute of Biomedical Sciences School of Physics and Electronics Shandong Normal University Jinan Shandong China; ^2^ Department of Radiation Oncology Shandong Cancer Hospital Jinan China

**Keywords:** cone beam CT, deformable image registration, hepatocellular carcinoma, radiation‐induced liver disease

## Abstract

Many patients with technically unresectable or medically inoperable hepatocellular carcinoma (HCC) had hepatic anatomy variations as a result of interfraction deformation during fractionated radiotherapy. We conducted this retrospective study to investigate interfractional normal liver dosimetric consequences via reconstructing weekly dose in HCC patients. Twenty‐three patients with HCC received conventional fractionated three‐dimensional conformal radiation therapy (3DCRT) were enrolled in this retrospective investigation. Among them, seven patients had been diagnosed of radiation‐induced liver disease (RILD) and the other 16 patients had good prognosis after treatment course. The cone‐beam CT (CBCT) scans were acquired once weekly for each patient throughout the treatment, deformable image registration (DIR) of planning CT (pCT) and CBCT was performed to acquire modified CBCT (mCBCT), and the structural contours were propagated by the DIR. The same plan was applied to mCBCT to perform dose calculation. Weekly dose distribution was displayed on the pCT dose space and compared using dose difference, target coverage, and dose volume histograms. Statistical analysis was performed to identify the significant dosimetric variations. Among the 23 patients, the three weekly normal liver D_50_ increased by 0.2 Gy, 4.2 Gy, and 4.7 Gy, respectively, for patients with RILD, and 1.0 Gy, 2.7 Gy, and 3.1 Gy, respectively, for patients without RILD. Mean dose to the normal liver (D_mean_) increased by 0.5 Gy, 2.6 Gy, and 4.0 Gy, respectively, for patients with RILD, and 0.4 Gy, 3.1 Gy, and 3.4 Gy, respectively, for patients without RILD. Regarding patients with RILD, the average values of the third weekly D_50_ and D_mean_ were both over hepatic radiation tolerance, while the values of patients without RILD were below. The dosimetric consequence showed that the liver dose between patients with and without RILD were different relative to the planned dose, and the RILD patients suffered from liver dose over hepatic radiation tolerance. Evaluation of routinely acquired CBCT images during radiation therapy provides biological information on the organs at risk, and dose estimation based on mCBCT could potentially form the basis for personalized response adaptive therapy.

## 1 Introduction

Radiotherapy has been an important treatment modality for patients who had an unresectable or inoperable terminal‐stage hepatocellular carcinoma (HCC).^1^ Meanwhile, image‐guided radiotherapy (IGRT) incorporated with the technique of respiratory‐control (e.g., Real‐time Position Management^™^ System, Varian Medical Systems, Palo Alto, CA, USA) and highly conformal radiotherapy has better locoregional control rate and survival rate for HCC.^2^

However, radiation‐induced liver disease (RILD) is one of the most severe radiation‐related complications for patients who undergo hepatic RT, which prevents radiation dose escalation and re‐irradiation for hepatobiliary malignancies.^3^ RILD typically occurs 4–8 weeks after RT completion, its clinical characteristics are manifested grossly as nonmalignant ascites, upper gastrointestinal hemorrhage, and veno‐occlusive, which resemble Budd–Chiari syndrome, and RILD is almost fatal since there is no effective treatment at present.^4^ Liang et al. conducted a retrospective study and reported that 19 of 128 (15%) patients were observed developed RILD over 4 weeks after hypo‐fractionated three‐dimensional conformal radiation therapy (3DCRT), while 85% of these RILD patients died from RILD despite receiving appropriate treatments.^5^ Xu et al. reported that 17 of 109 patients developed RILD with elevations of AKP, or sGOT, and sGPT appearing in all patients within 4 months after irradiation, and noted that 13 of 17 (76%) died after onset of RILD.^6^ Hence, a high mortality rate of RILD deserves special attention.

Liver is believed to be a typical parallel organ; the normal liver will escape from damage provided that an adequate normal liver volume is not irradiated to high doses.^7^ Liang et al. proposed D_mean_ for prediction of RILD, considering that liver received inhomogeneous dose and hepatic radiation tolerance had severe volume effect.^8^ In their study, D_mean_ of 23 Gy was estimated as the hepatic radiation tolerance for primary liver cancer patients with Child‐Pugh Grade A cirrosis treated with 3DCRT, which produced a high prediction rate (72%). Prevention of RILD by keeping dose to normal liver below the hepatic radiation tolerance is of predominant importance when designing treatment plan, whereas the planned dose is generally assumed, inconsistent with actual delivered dose.

Radiotherapy evolves toward more adaptive techniques. According to the routine adaptive radiotherapy (ART) strategy,^9^, ^10^ a repeat imaging scan to check whether it needs a re‐planning is implemented 2.5–3 weeks after the beginning of the treatment, whereas the time interval between the planning CT (pCT) and the repeat CT is too long to timely prevent the radiation overdose to the normal liver. Stewart et al. reported that the dose accumulation over weekly repeat magnetic resonance imaging (MRI) scans could be used to cope with the motion of the organ and tumor regression. Indeed, deformable image registration and dose accumulation can aid in evaluating the robustness of planning solutions on predicting the accumulated dose, whereas their approach without dose calculation is not reliable for practical clinical application.^11^ Moreover, the anatomic variations and dose distribution is unknown at each fraction until the repeat CT or MRI scan.

Image guidance plays an increasingly important role, not only in patient setup but also in monitoring the delivered dose and adapting the treatment to patient changes. Due to its superior soft tissue differentiation, ultrafast sequences, and the absence of ionizing radiation, MRI is an excellent candidate for real‐time image guidance in radiotherapy, and a 3D tracking method in 2D MRI series was developed for liver motion tracking and allows for real‐time 3D localization with MRI‐Linac systems.^12^, ^13^ The hybrid MRI and linear accelerator machines, which is able to compensate for patient anatomy changes, are currently under development, and MRI‐based radiotherapy planning will allow plan adaptation to the latest anatomy state in an online regime.^14^, ^15^ The online MRI‐Linac systems have potential feasibility of reducing the inter‐ and intrafractional anatomic changes induced excess radiation dose delivered to the patients. However, one major drawback of these methods is that they rely on state‐of‐the‐art technologies or treatments not commonly available to the majority of radiotherapy centers.

Currently, given the prevalence of cone beam CT (CBCT), imaging directly at the treatment position is convenient to correct photon therapy setup with the use of gantry‐mounted CBCT. CBCT can conveniently acquire volumetric images just before treatment with relatively low dose (~3 cGy).^16^ While hepatic anatomy variations are common due to the presence of the anatomic changes or body weight/habitus loss,^17^ this may lead to undesired radiation to the healthy parts of the liver. Yang et al. investigated that dose calculation based on modified CBCT (mCBCT) was more accurate than directly on the basis of CBCT.^18^ Landry et al. reported that dose distributions calculated on the modified CBCT agreed well to those calculated on the CT when using gamma index evaluation, as well as DVH statistics based on the same contours.^19^ This means that mCBCT has potential to account for interfractional dosimetric uncertainness.

This retrospective study was conducted to estimate the interfractional normal liver (the total liver minus GTV) dose consequences to the HCC patients, especially for RILD patients.

## 2 Materials and methods

### 2.A Patient data acquisition

A total of 23 patients who underwent 3DCRT with unresectable HCC were enrolled in this study. None of the patients, with absence of obstructive jaundice and uncontrollable ascites, had regional lymph node or extrahepatic metastases. According to the Child‐Pugh classification for liver cirrhosis, none of the patients presented with Child‐Pugh B or C classification of liver cirrhosis. Nine of them underwent transcatheter arterial chemoembolization (TACE) prior to irradiation, the interval between TACE and RT was about 3–5 weeks to allow the recovery of liver function, irrespective of which treatment was given first. Among 23 patients, 7 patients had been diagnosed of being RILD over 4 weeks after completion of RT (Table 1).

**Table 1:** The patients‐related characteristics in patients with and without RILD

Characteristic	No. of pts^a^ with RILD	No. of pts without RILD
Gender		
Female	2	4
Male	5	12
Age		
Range	47–63	32–66
TNM stage		
T2N0M0	1	10
T3N0M0	4	6
T4N0M0	2	0
PVT		
Present	4	6
Absent	3	10
TACE		
With	2	10
Without	5	6
NLV(cm^3^)	1031.6 ± 128.2	1418.6 ± 127.4

a Pts = patients.

### 2.B Treatment preparation

#### 2.B.1 Planning

For intravenous contrast planning fan‐beam CT (pCT) scans, all patients were immobilization with vacuum mold and the assistance of a tight abdominal belt to reduce the imaging artifacts using a Brilliance Big Bore CT simulator (Philips Inc., Eindhoven, Netherlands). The pCT scans were directly transmitted to the commercial treatment planning system (TPS) (Eclipse v13.5, Varian Medical Systems, Palo Alto, CA, USA).

For each of cases, four or five coplanar fields were designed to implement irradiation. The dose constraints for OAR were as follows: According to the clinical protocol reference of RTOG 0436 and Quantitative Analysis of Normal Tissue Effects in the Clinic (QUANTEC),^20^ mean dose to the normal liver (D_mean_) was limited to 28 Gy and D_50_ was limited to 35 Gy. The dose‐volume histogram (DVH) of normal liver was within the tolerance area (i.e., V_20_ < 49%, V_30_ < 28%, and V_40_ < 20%).^21^ For the stomach and duodenum, the maximum dose was limited to 45 Gy, and the volume receiving > 22.5 Gy was limited to < 5 cm^3^ (Table 2).^22^ It was also required that prescription dose cover at least 95% of the PTV and 100% of the GTV when D_mean_ was kept below 28 Gy. RT for HCC was delivered with linear accelerators (Varian Trilogy^™^) using either 6 MV or 15 MV X‐rays. The total dose range from 48–54 Gy, 2 Gy per fraction, and 5 fractions per week.

**Table 2:** Planned dose constraints

Structure	Dose constraints
Normal liver	D_mean_ < 28 Gy
	D_50_ < 35 Gy
	V_20_ < 49%
	V_30_ < 28%
	V_40_ < 20%
Stomach/duodenum	Max dose < 45 Gy
	V_22.5_ < 5 cm^3^

#### 2.B.2 CBCT imaging

Each patient has one initial pCT and 3 weekly CBCT. The CBCT images were taken with linac‐mounted On‐Board Imager (OBI, Varian Medical Systems, Palo Alto, CA, USA) in treatment position before dose delivery. The CBCT scans were acquired once weekly for the first 3 weeks. Thereafter, the re‐planning was executed to continue the rest of the treatment.

The CBCTs were acquired in half‐fan mode, full rotation, 120 kVp, 40 mA, 40 ms, with a maximum FoV of 45 cm in diameter, the collimators for kV X‐ray were limited to 10 cm in the patient superior–inferior direction to reduce the scatter effect on the CBCT images, and the imaging resolution was 0.879 × 0.879 × 1 mm^3^. As the CBCT images were acquired over a period of several respiratory cycles (approximately 2 min), resultant 3D image data provide an average position of the organs.

### 2.C Application of deformable image registration

#### 2.C.1 Algorithm

Because of the presence of inconsistent intensities between CT and CBCT, especially in chest and abdomen cases, intensity‐based deformable registration algorithms are susceptible to distorting the tissues, which would cause significant registration error.^23^, ^24^ Considering that large gradient generally exists on the border of organs or tissues, gradient field is not subject to the disparity of CBCT and pCT, gradient‐based deformable registration can effectively reduce the aforementioned registration error. The gradient‐based free form deformable registration (GFFD) algorithm was embedded in our in‐house developed software.

Bidirectional deformation vectors fields (DVFs) was computed to facilitate reconstruction of weekly dose and propagation of structural contours. Herein, we use forward DVFs to represent the CT‐to‐CBCT transformation and backward DVFs to represent the CBCT‐to‐CT transformation. The registration accuracy was validated in the previous work.^25^

#### 2.C.2 Reconstruction of weekly dose

**Figure 1:**
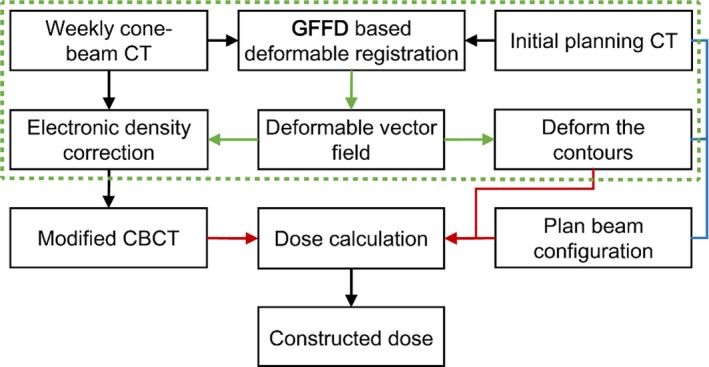
Modified cone‐beam CT‐based weekly dose reconstructed framework. The dashed box illustrates the application of GFFD deformable registration.

The method we proposed to reconstruct weekly dose while accounting for anatomic changes required the weekly CBCT and initial pCT, and the workflow consists of the following steps being illustrated in Fig. 1.

The liver motion following respiration and the gantry rotation made the CBCT images more prone to blur, and the deteriorated image quality hampered reliability of CBCT‐based dose calculation. In order to calibrate the Hounsfield Units (HU) values of CBCT in terms of electron density, the HU values of pCT was exploited instead of HU values of CBCT because pCT has a stable and reliable HU‐electron density relationship. The electronic density information was mapped from pCT to CBCT via DIR, and this process was repeated for each weekly CBCT available for the patient.

**Figure 2:**
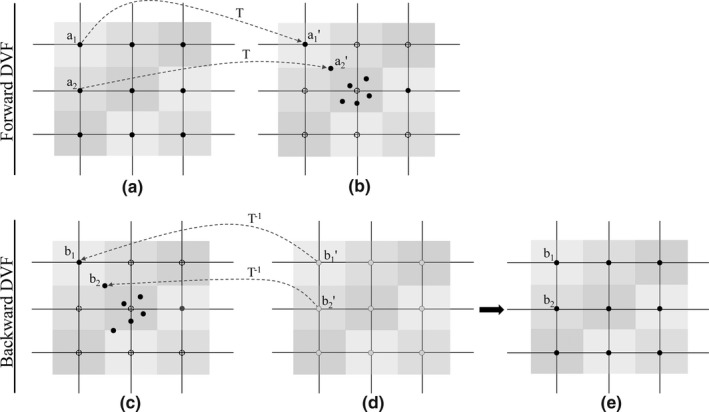
Illustration of the forward DVF and backward DVF. (a) Representation of the grid nodes (solid points) superimposed on the pCT, and the node was located on the center of each voxel, the different color was used to differentiate the different voxel. (b) Representation of the CBCT. Driven by the forward DVF, a perfect match of a_1_ and a_1_′ was accomplished, while a_2_ was not transformed to the corresponding node, but someplace nearby, e.g., a_2_′. The dotted nodes represented that no points were transformed to here. (c) pCT and (d) CBCT, driven by the backward DVF, nodes superimposed on the CBCT were transformed to the voxels of pCT. (e) mCBCT with HU value of pCT.

As seen from (Fig. 2), the grid nodes controlled the local deformation, and the nodes were located on the center of the voxel. The forward DVFs was derived from the DIR from pCT to CBCT, and the forward DVFs recorded where the HU value of the CBCT moved to the corresponding position of pCT. After computing the backward DVFs, the HU value of CBCT was updated by the HU value of pCT through the backward DVFs. The mCBCT contained the HU values of pCT while preserving the anatomic information of CBCT. Herein, the voxels of CBCT may not be able to match exactly through the forward DVF. Whereas the backward DVF could assist each CBCT voxel to find the corresponding HU value of pCT.

The weekly mCBCT were regarded as the pretreatment CBCT possessing HU values of pCT, and each scan was assumed to display the latest patient anatomy until the next scan. To reconstruct the weekly dose, the same beam configuration and dose constraint as the initial plan profiles recording were applied to the mCBCT‐based dose calculation process. The dose calculation algorithm employed in this study is collapsed cone algorithm.

#### 2.C.3 Propagation of structural contours

Due to the complex and highly deformable nature of organ and target motion, a simple rigid registration, guided by bone matching, is insufficient. To account for the interfraction anatomic changes, starting with rigid registration to align these two sets of images, the DIR was executed on every case. And the accuracy of the propagated contours was checked by the attending radiation oncologist.

### 2.D Dose assessment

The dose assessment was extended to examine the dosimetric impact within different structures, and DVH is a useful tool to assess if the plan is appropriate for the patient, by displaying in a quantified and comprehensive way the information of the dose delivered both to organs and targets. The DVHs for both manually drawn and mapped structures of interested were computed from the reconstructed dose distributions for each patient.

The liver V_20_, V_30_, V_40_, D_50_, and D_mean_ were evaluated, respectively. According to the findings of RTOG 0436 and QUANTEC, D_mean_ of 28 Gy and D_50_ of 35 Gy was used as reference of hepatic radiation tolerance in this study.

The symmetric DIR facilitated accounting for the geometric variations and ensured invertibility, and allowed the backward DVFs remap the weekly dose onto the pCT. Choosing the pCT as reference allowed to iteratively compare the planned objectives with the delivered ones as the treatment progresses. Hence, each reconstructed weekly dose of the same patient was mapped to pCT scan, and dose deviations (D_weekn_–D_plan_) displayed on pCT was exploited to investigate week‐by‐week dose difference.

### 2.E Statistical analysis

Statistical analyses were implemented using SPSS software (SPSS ver.19.0, Inc., Chicago, IL, USA). The independent‐samples t‐test was performed to identify the significant dosimetric changes.

## 3 Results

### 3.A Uncertainties of the anatomic variations

**Figure 3:**
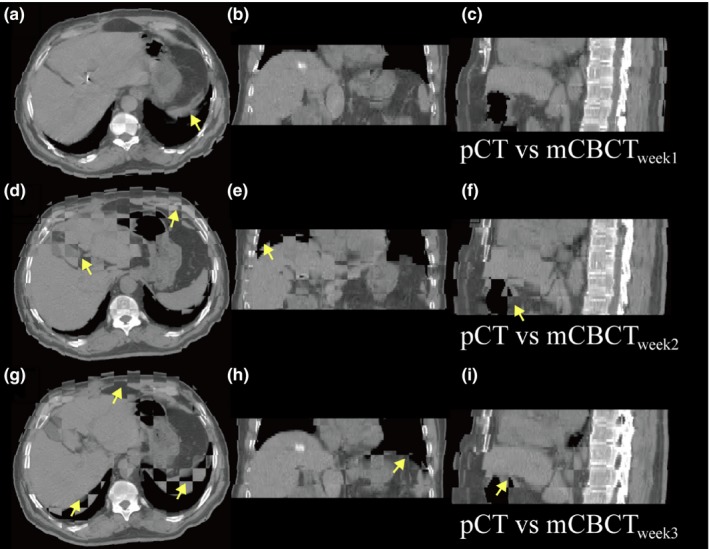
Checkerboard comparison between the pCT and mCBCT_weekn_ image before deformable registration, and the arrows indicate the visible differences.; (a), (b), and (c), the pCT vs mCBCT_week1_, (d), (e), and (f), pCT vs mCBCT_week2_, and (g), (h), and (i), pCT vs mCBCT_week3_.

Figure 3 shows the checkerboard of pCT and weekly mCBCT, and we highlighted areas of misalignment in the checkerboard images before GFFD registration with arrows. In particular, it notices that the misalignment always occurs in liver and lung areas, where larger motion and deformation exists. In addition, the shape and position of the liver of the patient varies on different treatment fraction, and mCBCT_week3_ shows the most distinct visible difference among these three set of weekly mCBCT.

**Figure 4:**
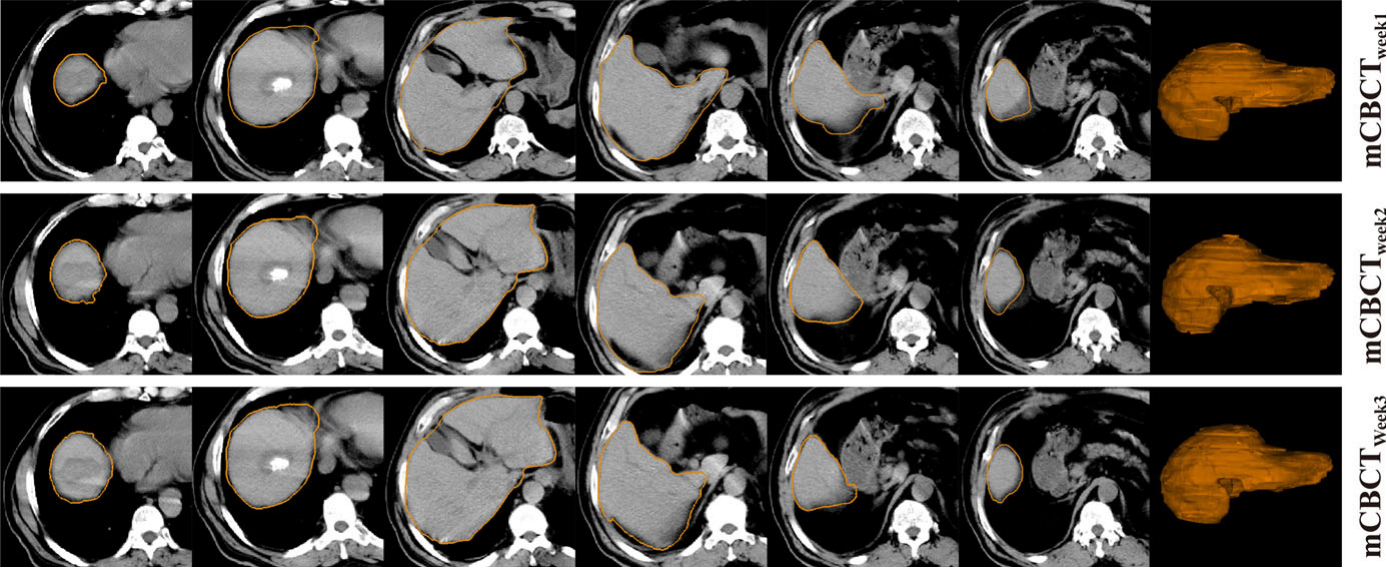
Example of slice and three‐dimension region for a sample segmented using deformable registration propagated methods for mCBCTweek1, mCBCTweek2, and mCBCTweek3. The subset of slices was arbitrarily selected by increasing the slice numbers to approximately cover the entire liver volume.

GFFD registration mapped the contours on CBCT from pCT to correct for the anatomic changes, and (Fig. 4) shows the interfractional liver shape results for a HCC patient. The subset of slices was arbitrarily selected by increasing the slice numbers to approximately cover the entire liver volume, and each column displayed the corresponding slice of the same patient.

### 3.B Comparison of dosimetric parameters

**Figure 5:**
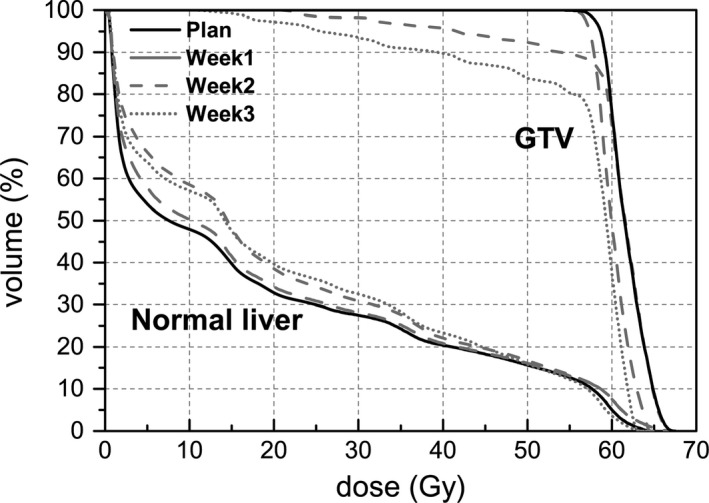
Illustration of DVH of normal liver and GTV for planning CT and three weekly mCBCT: mCBCT_week1_, mCBCT_week2_, and mCBCT_week3_. The DVHs calculated based on structural contours propagated by the DIR.

Weekly dose was extended to the entire fraction and compared with the planned dose, and an example of patient without RILD was shown in Fig. 5. The clinically significantly differences were observed in the high‐dose region for the GTV, the week2 and week3 DVH curves illustrated that the prescribed dose was unable to cover the entire GTV, and the normal liver suffered increased dose.

**Figure 6:**
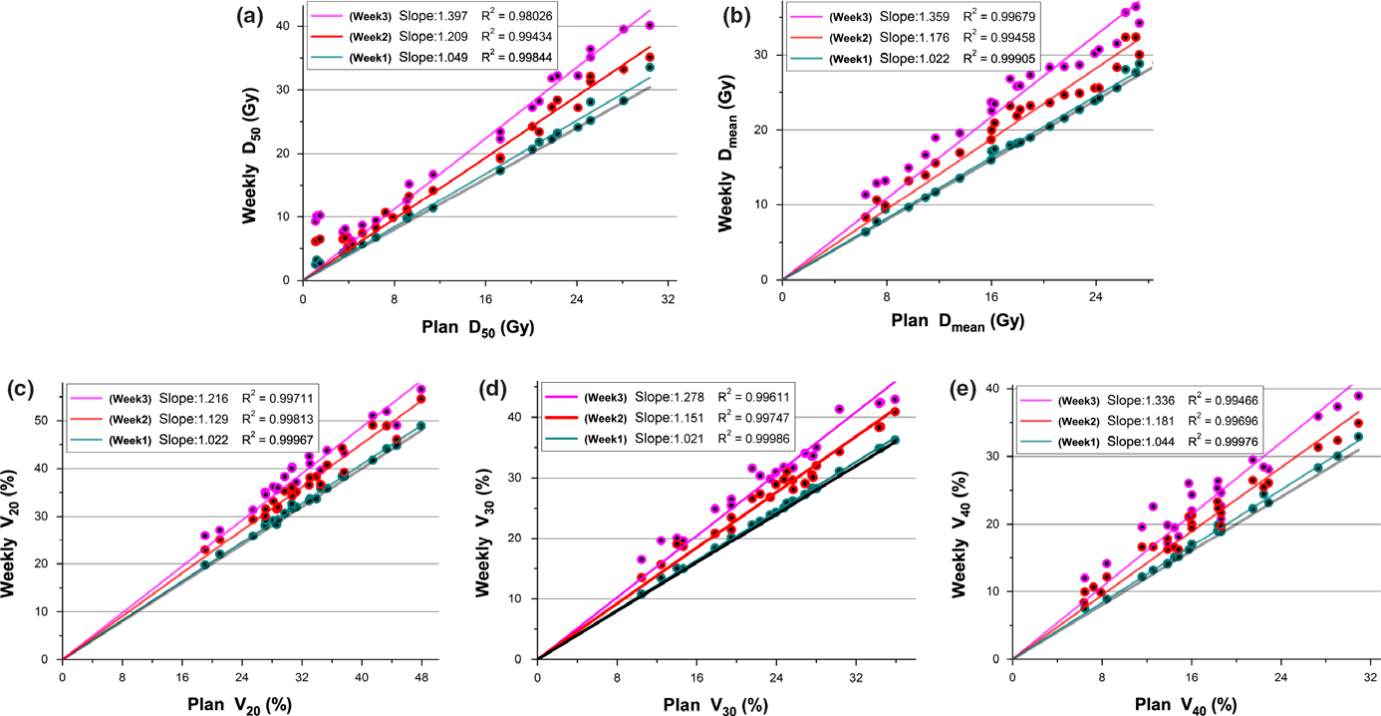
Correlations for the weekly (a) D_50_, (b) D_mean_, (c) V_20_, (d) V_30_, and (e) V_40_ of liver for plans calculated using pCT and weekly mCBCT. Dots above the line *y = x* (indicated by the black line) are reconstructed increases, dots below the line are reconstructed decreases. The slopes of the linearly fitted lines for D_50_, D_mean_, V_20_, V_30_, and V_40_ were computed and given in the top left of the plot, and the trend is measured here using the slope value.

As shown in Fig. 6, the ideal slopes of the linearly fitted lines for these dosimetric value was 1.0, respectively, while the slope of the reconstructed weekly dose exceeded 1.0 with an increased trend. This result demonstrated that the weekly dose values were increased on average by 4.9%, 20.9%, and 39.7% for D_50_, 2.2%, 17.6%, and 35.9% for D_mean_, 2.2%, 12.9%, and 21.6% for V_20_, 2.1%, 15.1%, and 27.8% for V_30_ and 4.4%, 18.1%, and 33.6% for V_40_, respectively.

**Figure 7:**
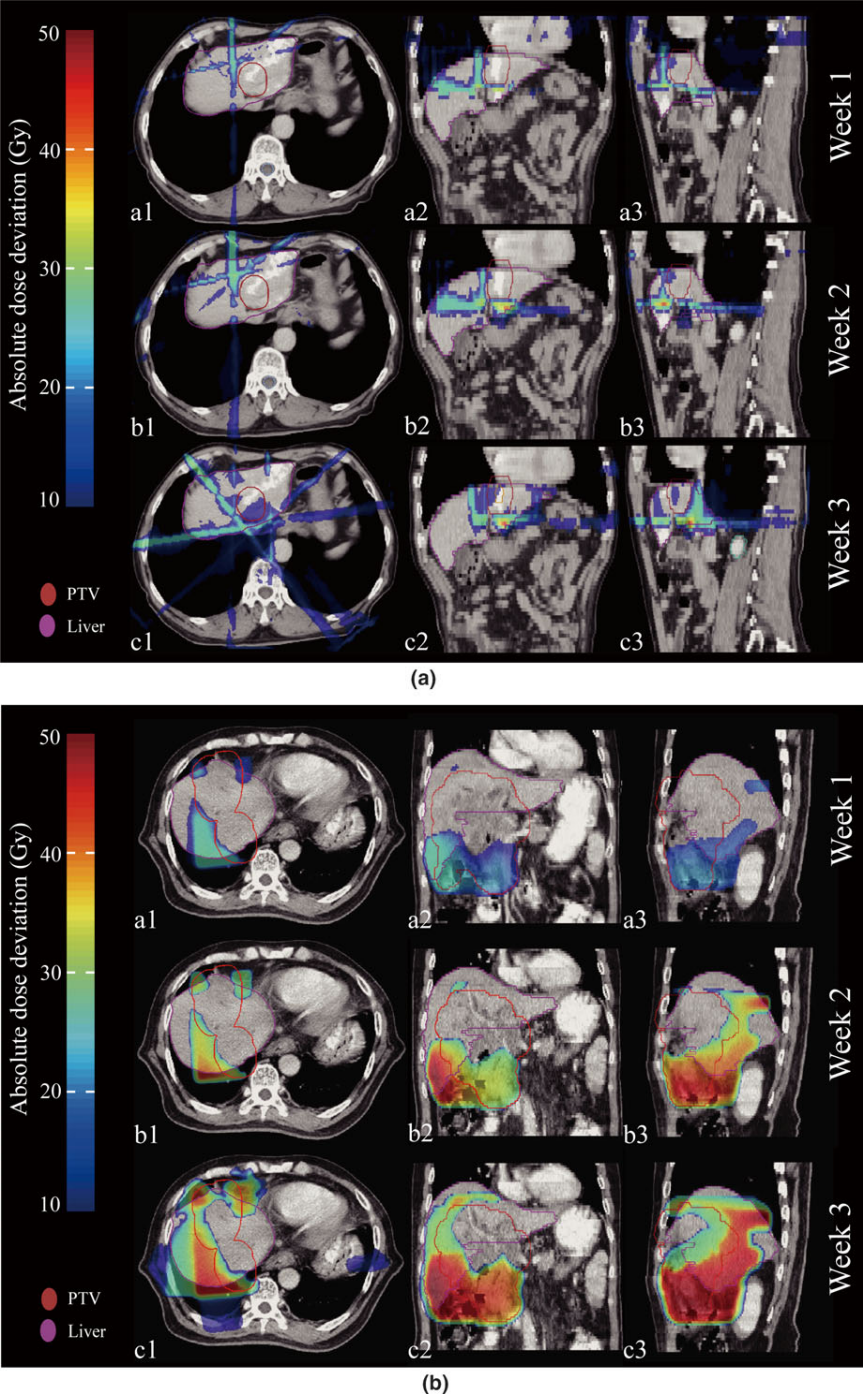
Example of absolute dose deviation on pCT for patients without (a) and with (b) RILD. The dose deviation between pCT and mCBCT was shown in three different periods (top for week1, middle for week2 and bottom for week3), and a fractional reconstructed dose deviation from the planned dose (D_mcbct_–D_plan_) are shown in the transverse (a1, b1, and c1), coronal (a2, b2, and c2), and sagittal (a3, b3, and c3) views, respectively. Each mCBCT was deformed to the pCT for dose subtraction.

Figure 7 shows examples of absolute dose deviations displayed on the pCT space. Fig. 7(a) shows a non‐RILD case while Fig. 7(b) shows a RILD case, and the range of dose deviation was expanded week by week in both cases, and the degree of dose deviation of RILD patient was observed higher than non‐RILD patient.

### 3.C Dosimetric parameters correlated with RILD

Upon univariate analysis, D_50_, D_mean_, and V_30_ were identified as significant dosimetric parameters related with RILD (all *P* < 0.05), and patients with RILD received higher D_50_ and D_mean_ than did those without RILD. Neither the V_20_ and V_40_ contributed to the risk of developing RILD (Table 3).

**Table 3:** The comparison of planned normal liver dosimetric parameters in patients with RILD and without RILD

Parameter	Patients with RILD (n = 7)	Patients without RILD (n = 16)	*P* value
V_20_ a(%)	40.1 (30.7–43.3)	29.3 (19.1–35.7)	0.086
V_30_ (%)	29.2 (22.1–34.3)	21.3 (10.5–25.7)	0.048
V_40_ (%)	22.9 (15.7–27.3)	15.4 (6.6–22.9)	0.086
D_50_ b(Gy)	26.2 (21.8–30.4)	13.6 (3.7–23.7)	0.001
D_mean_ (Gy)	25.3 (22.7–27.4)	17.7 (6.4–24.6)	0.011

a V_20_ = the percentage of normal liver volume that received ≥20 Gy in the total normal liver volume. The other V with suffixes express the same meaning, but the suffix numbers represent the doses received.

b D_50_ = dose to the 50% of the normal liver.

**Figure 8:**
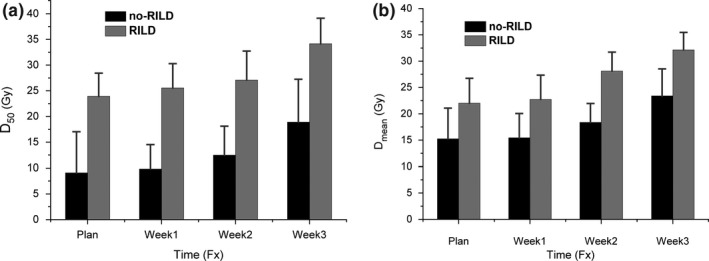
The reconstructed weekly (a) D_50_ and (b) D_mean_, relative to planned D_50_ and D_mean_. Error bars indicate standard deviation. Herein, no‐RILD represents patients who did not develop RILD post‐RT, and RILD represent patients were diagnosed of RILD post‐RT.

The reconstructed weekly dose between patients with RILD and without RILD were compared in Table 4. As for the patients with RILD, the D_mean_ of week2 were found higher than reference value (D_mean_ = 28 Gy), and D_50_ of week3 were found higher than reference value (D_50_ = 35 Gy). As for the patients without RILD, the reconstructed weekly D_50_ and D_mean_ were below the reference value. And it also showed that reconstructed weekly dose increased week by week (see Fig. 8). Furthermore, the increase in normal liver dose differed significantly between patients with RILD and without RILD, herein, the increase in normal liver dose was computed by taking the weekly dosimetric value minus planned one. Only V_30_ showed significant difference between patients with and without RILD in week1 (*P* < 0.05), while V_20_, V_30_, D_50_, and D_mean_ showed significant difference in week2, respectively, and V_40_ and D_50_ showed significant difference in week3, respectively.

**Table 4:** The comparison of normal liver dosimetric parameters in patients with RILD and without RILD among week1, week2, and week3

Parameter	Ref^b^	Pts^a^ with RILD (n = 7)			Pts without RILD (n = 16)			*P* * [Link]		
		Week1	Week2	Week3	Week1	Week2	Week3	Week1	Week2	Week3
V_20_ c (%)	*NA*	40.9 (32.6–44.1)	45.6 (36.1–48.9)	48.8 (40.0–51.9)	29.9 (19.7–36.6)	33.1 (22.9–41.2)	36.4 (25.5–44.3)	0.521	0.010	0.227
V_30_ (%)	*NA*	29.7 (23.2–34.8)	33.8 (27.1–38.2)	36.5 (30.1–42.3)	21.9 (10.8–26.1)	24.7 (13.4–28.6)	28.2 (16.5–31.8)	0.111	0.008	0.503
V_40_ (%)	*NA*	24.1 (16.6–28.3)	26.9 (21.3–31.2)	31.0 (24.2–35.9)	16.4 (7.5–21.5)	18.6 (9.6–22.2)	21.1 (11.6–24.2)	0.025	0.074	0.002
D_50_ d (Gy)	35	26.4 (22.3–31.5)	30.6 (27.3–33.1)	35.3 (31.8–38.1)	14.6 (4.5–24.8)	17.3 (6.7–25.4)	20.4 (8.1–30.2)	0.754	0.003	0.002
D_mean_ (Gy)	28	25.8 (23.5–27.3)	28.4 (24.9–32.3)	32.4 (28.6–36.1)	18.1 (6.7–25.3)	21.2 (8.4–27.6)	24.6 (11.3–31.4)	0.403	0.020	0.107

a Pts = patients.

b Ref = hepatic radiation tolerance.

c V20 = percentage of the normal liver volume that received exceeding 20Gy in the total normal liver; V30, V40 express the resembling meaning, except for the suffix numbers, which represent the doses received in increments of 10 Gy.

d D50 = dose to 50% of the normal liver.

*Significance in independent sample *t*‐test with *P* value less than 0.05 from two‐sided tests.

## 4 Discussion

Radiotherapy is commonly used to treat HCC at present, whereas RILD is a recognized life‐threatening complication, occurred more frequently as the dose to liver increased. With the advent of highly conformal radiation treatment planning and delivery technology, tumoricidal doses could be delivered safely provided that the mean dose to the normal liver was limited to below hepatic irradiation tolerance.^26^ In this study, that 7 of 23 (30.4%) patients had been diagnosis of RILD after RT does not represent the clinical incidence rate of RILD.

In addition, pioneering investigators has demonstrated that the severity of hepatic cirrhosis is the most significant independent clinical predictor for RILD, and more RILD cases are expected to occur in patients with Child‐Pugh Grade B or C hepatic cirrhosis;^27^ the acknowledged rationale is that the severely cirrhotic hepar is less tolerable to the irradiation of X‐ray due to fact that cirrhosis hinders the repair of radiation injury along with the hepatocyte proliferation.^28^ Thus, the baseline hepatic function status is an important factor for the occurrence of RILD as a limited number of researchers have found. In order to eliminate the confounding factor, patients with Child‐Pugh Grade B or C were excluded from our study while those exclusively with Child‐Pugh Grade A were eligible.

In this work, we used CBCT‐based dose constructed methods to estimate irradiation dose to the normal liver, using data from weekly CBCT. CBCT images include larger amounts of scattering than pCT, resulting in larger variation in HU values that limit the HU calibration and reliability. The CBCT correction is done via overriding the HU values of CBCT with HU values of pCT. The mCBCT‐based dose reconstructed method presented allows for acceptable dosimetric evaluation, comparable to doses recalculated on pCT, and this had been validated by Yang et al.^18^

Importantly, however, spatial analysis of IMRT high‐ and low‐dose PTV volumes indicated that consistent regions of the PTV are being under‐ or over‐dosed. This may have a clinically detrimental effect for individual patients, e.g., if a patients’ tumor was adjacent to the chest wall, where low‐dose regions are typically located. In such situation, re‐planning would be appropriate, emphasizing the need for spatial isodose analysis to allow suitable adaptation.

The clinical outcomes of a small‐margin approach with frequent re‐planning strategy are unclear. Recent work on the dosimetric consequences of PTV margin size confirms that as the PTV margin decreases, accumulated OAR doses decrease, at the expense of an increased risk of target underdosing.^29^ The re‐planning strategy outlined in this study mitigated this risk of target miss and preserved the OAR dose sparing gains of small‐margin IMRT for the population as a whole.

Note that the dose objectives of target and OARs may not be simultaneously met, and the loco‐regional tumor control should be emphasized at the expense of OAR dose sparing. The anatomical change resulted in liver dose increase relative to planned dose week by week (Fig. 7), and the dose difference was increased week by week (Fig. 8). Although the planned dosimetric parameters were below the hepatic radiation tolerance, the planned one determined the baseline of the liver dose without considering the interfraction dose change. In this case, those patients who were diagnosed with RILD after RT were at the higher risk of liver overdose. Furthermore, Table 4 showed that the normal liver dosimetric value had a significant increasing trend from week1 to week2, and the liver overdose was observed for patients with RILD. If adaptive re‐planning was implemented as soon as possible, and the delivered liver dose was ensured below the hepatic radiation tolerance per fraction, the liver overdose would be avoided in most circumstances.

In the presence of interfraction anatomical changes, accurate knowledge of doses distribution would potentially reduce RILD risks and ensure target dose escalation. Automatic contour propagation, one of the applications of deformable registration, is beneficial to account for interfractional geometric changes, and dose calculation applying the plan beam profiles is capable of acquiring the interfractional dosimetric variables like V_20_, V_30_, V_40_, D_50_, and D_mean_. Incorporation with better knowledge of the reconstructed dose distribution, oncologists could assess the prospective risk of RILD in a timely manner. The automatic contour propagation has relations with DVH results. In this study, the verifications for deformable registration is to check how well the automatically generated contours match the structures in the mCBCT image, and visual contour verification by expert oncologists is a direct way to qualitatively evaluate the deformable registration while there is no good gold standard available.

This study is not without limitations and assumptions. The primary limitation is that the weekly CBCT scans were extrapolated to 24–27 fractions, which depended on the fractionation scheme, and each scan was assumed to represent the patient anatomy until the next scan. We are currently in the process of recruiting patients to have CBCT scans performed three times weekly to validate this study. This study also assumed perfect bone to bone matching to achieve zero setup errors. Furthermore, due to the respiratory and liver motion during CBCT scan, the CBCT indicates an intricate effect of multiple respiratory phase.^30^ Moreover, the often significant intra‐fraction motion^31^ was assumed to be zero in this study. This may have further underestimated the dosimetric consequences of random anatomical variations.

## 5 Conclusions

In this work, we proposed to deform the planning CT to CBCT for generating a reconstructed dosimetry to avoid the inaccuracies related to the inherent CBCT artifacts. Through the weekly reconstructed liver dose, patients with RILD were found over hepatic radiation tolerance before re‐planning, and patients without RILD were found below the tolerance. The modified CBCT may be a useful treatment monitoring and plan adaptation decision making tool for centers equipped with gantry mounted CBCT scanners. While more rigorous testing of this method might be necessary before clinical implementation. This study found mCBCT‐based dose reconstruction to be a potentially feasible method for routine dose monitoring during the course of radiotherapy.

## Conflicts of Interest

The authors have no relevant conflicts of interest to disclose.
